# Toll-like receptor 2 mediates *Acanthamoeba*-induced allergic airway inflammatory response in mice

**DOI:** 10.1371/journal.pntd.0011085

**Published:** 2023-01-27

**Authors:** Mi-Kyung Park, Hye-Kyung Park, Hak Sun Yu

**Affiliations:** 1 Department of Parasitology and Tropical Medicine, School of Medicine, Pusan National University, Yangsan, Republic of Korea; 2 Department of Internal Medicine, Pusan National University Hospital, Busan, Republic of Korea; 3 Research Institute for Convergence of Biomedical Science and Technology, Pusan National University Yangsan Hospital, Yangsan, Republic of Korea; Georgetown University, UNITED STATES

## Abstract

**Background:**

Repeated intranasal exposure to *Acanthamoeba* has been revealed to induce allergic airway inflammatory responses in mice. Based on the role of toll-like receptors (TLRs) in the pathogenesis of allergic asthma, TLRs form a link between innate and adaptive immune responses, and play an important role in the activation of various cells in the innate immune system.

**Methodology/principal findings:**

To determine the TLRs that are related to these immune responses, we assessed the expression levels of inflammation-related genes in mouse lung epithelial (MLE)-12 cells treated with excretory-secretory proteins (ES-P) of the *Acanthamoeba* strain (KA/E2) with or without the TLR antagonists. The expression levels of inflammation-related genes, such as eotaxin, TARC, macrophage-derived chemokine (MDC), and TSLP, in the TLR2 and TLR9 antagonist treatment groups were decreased, compared to those in the ES-P alone or other TLR antagonist treatment groups. In particular, a greater decrease in the relevant gene expression levels was found in the TLR2 antagonist treatment group than in the TLR9 antagonist treatment group. Allergic airway inflammation was evaluated in the wild-type (WT) and TLR2 knockout (KO) groups following KA/E2 exposure. Based on the results, allergic airway inflammatory responses (airway resistance value, inflammatory cell infiltration, Th2-related cytokine expression, mucin production, and metaplasia of lung epithelial cells and goblet cells) by KA/E2 were reduced in the TLR2 KO groups. In addition, TLR2 knockout BMDCs displayed lower activation of surface markers owing to ES-P stimulation than normal BMDCs, and KA/E2 ES-P–treated TLR2-depleted BMDCs produced fewer Th2 cytokine-expressing cells from naïve T cells than WT BMDCs. When ES-P was administered after primary lung cells were obtained from WT and TLR2 KO mice, the expression levels of inflammation-related genes were found to be significantly decreased in TLR2 KO cells compared to those in WT cells.

**Conclusions:**

These results suggest that TLR2 is involved in lung inflammatory response activation in KA/E2 intranasal infection, especially in airway tissue.

## Author summary

TLRs can recognize and bind to several PAMPs and trigger a series of signal transductions, ultimately leading to the release of inflammatory mediators, initiating the innate immune response, removing the invasive pathogenic microorganisms, and playing an important role in innate immune defense. According to many recent studies, TLR2 plays an important role in allergic diseases, such as asthma; however, its mechanism has not been fully revealed. Here, we demonstrated the interaction between the KA/E2 infection and TLR2 in vivo and in vitro. Although our findings provide invaluable insights into the mechanisms of the interaction between parasites and host, the mechanisms whereby KA/E2 infection induces allergic airway inflammation in the host should be clarified via further research. Overall, in this study, mice with TLR2 gene knockout were used as the study objects and KA/E2 trophozoites were induced as an allergen to establish the asthmatic mouse model. The role of TLR2 in the asthmatic mouse model and its possible mechanism of action were observed, thereby providing new insights into the pathogenesis of asthma and identifying new possible targets for asthma prevention and treatment.

## Introduction

Allergic asthma involves T and B lymphocytes, eosinophils, basophils, mast cells, and recently discovered type 2 innate lymphocytes, which cooperate in its pathological mechanism. Allergic asthma is caused by the inhalation of various allergens, including components from house dust mites, pollen, plants, parasites, molds, and pets [1–4]. There are 300 million patients with asthma worldwide, and the number of patients is increasing annually owing to the increased abundance of allergens caused by global warming and changes in the living environment [1,2].

*Acanthamoeba*, a pathogenic and opportunistic free-living amoeba, is a protozoan genus that can survive in various environments and is isolated from soil, dust, air, water seawater, swimming pools, domestic tap water, and contact lenses [5,6]. Repeated intranasal exposure to *Acanthamoeba* trophozoites induces allergic airway inflammation in vivo [3]. Patients with positive results for *Acanthamoeba* in the skin-prick test response exhibited higher *Acanthamoeba*-specific IgE levels than other patients and healthy persons [7]. Our previous studies revealed that KA/E2 may be a novel allergen.

The Toll-like receptor (TLR) family member, TLR2, is the most widely expressed TLR and exists extensively in plants, insects, mammals, and humans. TLR2 has been demonstrated to sense pathogen-associated molecular patterns (PAMPs) from a wide range of viruses, phyla, bacteria, fungi, parasites, and inflammation-induced damage-associated molecular patterns of self-origin [8–11]. This wide breadth of pathogen recognition is partly due to its unique ability to heterodimerize with other members of the TLR1 superfamily (e.g., TLR1, 6, and 10) and non-TLR cellular molecules [12,13]. TLR2 is a type I transmembrane protein that, as a pattern recognition receptor, can recognize and bind to several PAMPs and trigger a series of signal transductions, thereby leading to the release of inflammation mediators, initiating the innate immune response, removing invasive pathogenic microorganisms and playing an important role in innate immune defense [12,13]. Furthermore, many studies, including a study from 2017, reported that TLR2 plays an important role in allergic diseases, such as asthma; however, the mechanism has not been fully elucidated [14].

In this study, mice with TLR2 gene knockout were used as objects and inoculated with KA/E2 trophozoites as an allergen to establish the asthmatic mouse model. The role of TLR2 in an asthmatic mouse model and its possible mechanism of action were assessed to provide new insights into the pathogenesis of asthma and identify new possible targets for its prevention and treatment.

## Materials and methods

### Ethics statement

All animal experiments were approved by the Pusan National University School of Medicine Animal Care and Use Committee (Approval No. PNU-2013-0260). Mice were divided into five groups.

### *Acanthamoeba* cultivation and excretory-secretory proteins (ES-P) preparation

The KA/E2 strain had the same molecular characteristics as the *A*. *lugdunensis* L3A strain (ATCC 50240) [15]. ES-P was prepared as previously described [3]. To obtain ES-P, the trophozoites were incubated in PYG medium for 3–5 days at 25 °C. Following freeze-drying (at 50 mTorr and -80 °C for 20 h), excessive salts were eliminated from the concentrated medium using HiTrap Desalting (GE Healthcare, Uppsala, Sweden). Thereafter, lipopolysaccharide was depleted (endotoxin levels <0.01 μg/ml) from the ES proteins using Detoxi-Gel Affinity Pak pre-packed columns (Pierce, Rockford, Illinois, USA), in accordance with the manufacturer`s instructions.

### In vitro stimulation to analyze inflammation-related gene expression

Mouse lung epithelial cells (MLE12 cells) were obtained from ATCC (Manassas, VA, USA). MLE12 cells were seeded at a density of 4 × 10^5^ cells in 24-well plates and incubated overnight at 37 °C. Thereafter, the cells were stimulated with ES-P for 3 h with or without pretreatment with the TLR1/2 (Sigma-Aldrich, 614305), TLR4 (Sigma-Aldrich, P4932), TLR5 (MedChemExpress, TH1020), TLR7/8 (Miltenyi Biotec, 130-105-819), or TLR9 (Invivogen, ODN 2088) antagonists. MLE12 cells were collected in 1 mL of QIAzol (Qiagen, Hilden, Germany), and RNA extraction was performed in accordance with the manufacturer’s protocols for the transcription of 2 μg of RNA. Real-time PCR was performed using an iCycler (Bio-Rad, Hercules, California, USA) to determine the mRNA levels of interleukin (IL)-25, CCL11 (eotaxin), MDC, and thymic stromal lymphopoietin (TSLP). The following primers were used for real-time PCR: IL-25 (F-5′-TGG CAA TGA TCG TGG GAA CC-3′, R-5′-GAG AGA TGG CCC TGC TGT TGA-3′); Eotaxin (F-5′-GCG CTT CTA TTC CTG CTG CTC ACG G-3′, R-5′-GTG GCA TCC TGG ACC CAC TTC TTC-3′); MDC (F-5’-AAG ACA GTA TCT GCT GCC AGG-3’, R- 5’-GAT CGG CAC AGA TAT CTC GG-3’); and TARC (F-5′-AGA GCT GCT CGA GCC ACC AAT GTA-3, R-5′-CAC CAA TCT GAT GGC CTT CTT CAC-3′).

### Mice and experimental scheme

Female C57BL/6 mice (6 weeks old) were purchased from Samtako Co. (Gyeonggi-do, Korea), and TLR2 knockout (KO) C57BL/6 female mice were supplied by Pohang University of Science and Technology (Gyeongsangbuk-do, Korea). Mice in the positive control group were intranasally (IN) administered 5 × 10^4^ KA/E2 trophozoites six times at 2-day intervals. At 24 h after the last challenge, airway responsiveness was evaluated by measuring the change in lung resistance in response to aerosolized methacholine (Sigma-Aldrich). To measure bronchoconstriction, the enhanced pause was measured at baseline (phosphate-buffered saline aerosol; control) and after exposure to increasing doses of aerosolized methacholine (0–50 mg/mL) using whole-body plethysmography (Allmedicus, Korea). In the plethysmography procedure, mice were allowed to acclimate for 3 min, exposed to nebulized saline for 10 min, and treated with increasing concentrations (0, 12.5, 25, and 50 mg/mL) of methacholine using an ultrasonic nebulizer (Omron, Japan). After each nebulization, the enhanced pause values were measured every 150 s during the experimental period and then averaged. Graphs were generated, revealing the enhanced pause values in response to increased methacholine concentrations for each dose-matched group of mice.

### Analysis of bronchoalveolar lavage fluid (BALF) and differential cell counting

To obtain BALF, the tracheas of anesthetized mice were exposed and cut immediately below the larynx. A polyurethane flexible tube (BD Biosciences, San Jose, California, USA) attached to a blunt 24-gauge needle was placed in the trachea, and the lung was lavaged once with 800 μL of sterile phosphate-buffered saline. BALF samples were centrifuged for 5 min at 3,000 rpm and 4 °C. The supernatants were decanted and immediately frozen at −70 °C. The cell pellets were resuspended and washed twice with phosphate-buffered saline. The total cell numbers were counted using a hemocytometer. To determine the differential cell counts, BALF cells were prepared using a cytospin apparatus and stained with Diff-Quik solution (Sysmex Co., Kobe, Japan) in accordance with conventional morphological criteria. At least 200 cells per slide were evaluated to determine the differential leukocyte counts.

### Lung histopathology

Histological analyses were conducted as previously described [16]. Briefly, the lung tissues were fixed with formaldehyde and embedded in paraffin. Thin sections of the embedded tissues were stained with hematoxylin and eosin and periodic acid-Schiff and examined microscopically.

### Preparation of lymphocytes

After killing, the lung-draining lymph nodes were detached from mice. The tissues were then disrupted and treated with ACK hypotonic lysis solution (Sigma-Aldrich, USA) for 5 min at 24 °C for red blood cell lysis. After red blood cell lysis, the remaining cells were filtered through a 100 μm mesh (Small Parts, Inc. Miramar, USA), and the cells were seeded on 24-well plates at approximately 1 × 10^6^ cells/mL in RPMI 1640 supplemented with 10% fetal bovine serum (FBS) and penicillin/streptomycin. For the CD3 stimulation experiments, 0.5 μg/mL of the CD3 antibody (BD Pharmigen, BD Bioscience, USA) was added to the wells of the plate. After incubation, the culture medium was harvested and stored at −20 °C for enzyme-linked immunosorbent assay (ELISA).

### ELISA

The levels of IL-4, IL-5, IL-13, and IFN-γ in the CD3-stimulated lymphocyte culture supernatants and BALF were evaluated using ELISA kits (eBioscience, San Diego, CA, USA), according to the manufacturer’s protocol.

### BMDC isolation, culture, and stimulation

Bone marrow cells were flushed from the femurs and tibias of 7-week-old C57BL/6 wild-type (WT) and TLR2 KO mice, washed, and cultured in complete RPMI 1640 medium containing 10% heat-inactivated FBS, 50 μM 2-mercaptoethanol, 2 mM glutamine, and penicillin and streptomycin (100 U/mL and 100 μg/mL, respectively; Invitrogen, Carlsbad, CA, USA), and recombinant mouse granulocyte macrophage colony-stimulating factor (GM-CSF) and recombinant mouse IL-4 (10 ng/mL each; R&D Systems, Minneapolis, MN, USA). Non-adherent granulocytes were removed after 24 h of culture and fresh complete medium was added every other day. All cultures were incubated at 37 °C in a 5% CO_2_ atmosphere. On day 7, the cells were recovered and CD11c^+^ cells were isolated using magnetic beads. The sorted CD11c^+^ cells were used in further studies. Immature BMDCs were cultured in 6-well plates at a density of approximately 5 × 10^6^ cells/well in 3 mL of complete RPMI 1640 medium supplemented with 10% FBS and 40 ng/mL GM-CSF. BMDCs were stimulated with ES-P for 24 h.

### Co-culture of BMDCs with naïve T cells

BMDCs from WT and TLR2 KO mice were stimulated with KA/E2 ES-P and then incubated with naïve T cells. Th2 differentiation was analyzed using FACS and ELISA. The BMDCs (100 μL) were seeded in round-bottom wells of 96-well culture plates at approximately 2 × 10^5^ cells/well and stimulated with 5 μg/mL ES-P for 24 h at 37 °C. Naïve T cells (CD44^low^CD62L^high^CD25^−^) were isolated from WT mice using a cell sorter (BD Biosciences, San Jose, CA, USA). The isolated naïve T cells were co-cultured with BMDCs at a BMDC:T cell ratio of 1:5 for 72 h at 37 °C. Subsequently, for the CD3 stimulation experiments, 0.5 μg/mL of CD3 antibody was added to the cells and incubated for 72 h at 37 °C. ELISA was conducted to determine the levels of IFN-γ, IL-4, IL-5, IL-10, and TGF-β in the supernatants of ES-P- or CCR7 antibody–treated BMDCs and naïve T cells using an ELISA kit (eBioscience, San Diego, CA, USA), according to the manufacturer’s protocol.

### Primary lung cell isolation and stimulation

The lungs were extracted from WT and TLR2 KO mice, and the Mouse Lung Dissociation Kit (Miltenyi Biotec Inc., Bergisch Gladbach, Germany) and MACS were used to isolate primary lung cells from lung cell suspensions. All procedures for handling mice were performed according to the manufacturer’s protocol. WT- or TLR2 KO–primary lung cells (4 × 10^5^) were seeded in 24-well plates and incubated overnight at 37 °C. The cells were then stimulated with ES-P for 3 h. After stimulation, RNA was extracted from the cells with 1 mL of QIAzol (Qiagen, Hilden, Germany) and processed according to the manufacturer’s protocols for the transcription of 2 μg of RNA. Real-time PCR was performed using an iCycler (Bio-Rad, Hercules, California, USA) to determine the mRNA levels of IL-25, eotaxin, TSLP, and MDC.

### Statistical analysis

All data were analyzed using Prism 6 (GraphPad Prism, La Jolla, CA, USA). Mean ± SD was calculated, and significant differences were determined using the Student’s *t*-test or one-way ANOVA with the Dunnett’s multiple comparisons post-test for the comparison of all groups with the control group.

## Results

### MLE12 cells respond to TLR2 antagonists

**Fig 1:**
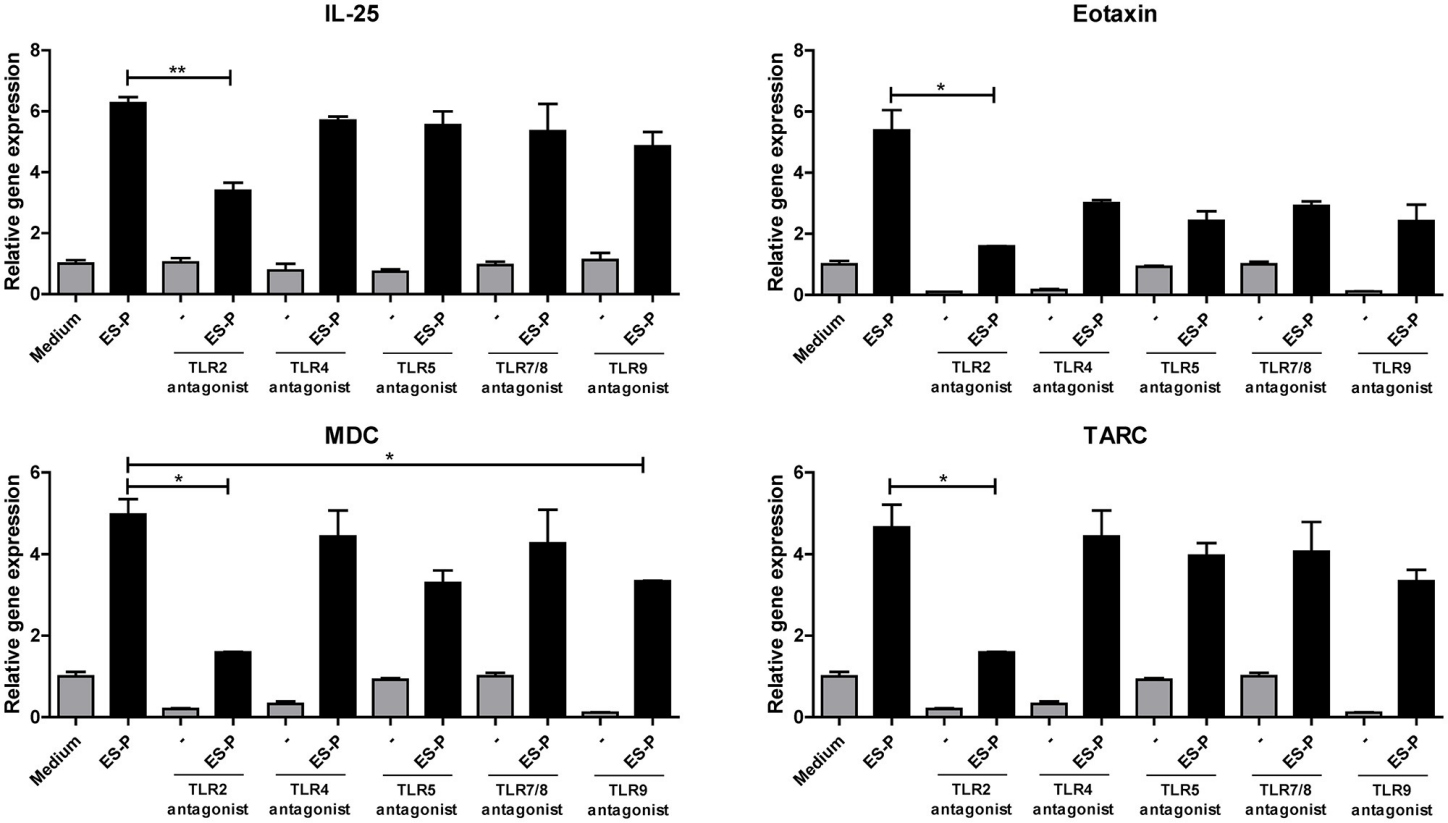
KA/E2 ES-P induced inflammatory-related gene expression by TLR2 in MLE12 cells. Comparison of the expression of inflammatory response-related genes in MLE 12 cells from the KA/E2 ES-P group or TLRs antagonist pre-treated group. (n = 3/group, 3 independent experiments, *; *p* < 0.05, **; *p* < 0. 01, ***; *p* < 0.001).

To assess the functionality of TLRs, we examined the responses of MLE12 cells to TLRs characterized by KA/E2 ES-P. We checked the inflammation-related gene expression level following ES-P treatment based on the presence or absence of the various TLR antagonist pretreatments. The expression levels of pro-inflammatory genes, including IL-25, eotaxin, TSLP, and MDC, were downregulated in MLE12 cells in response to treatment with the TLR2 and TLR9 antagonists compared to treatment with ES-P alone or the other TLRs antagonist (Fig 1). In particular, differences in the gene expression levels of the TLR2 antagonist group were more significant than those of the TLR9 antagonist group (Fig 1).

### TLR2 knockout alleviates allergic airway inflammation via KA/E2 exposure in vivo

**Fig 2:**
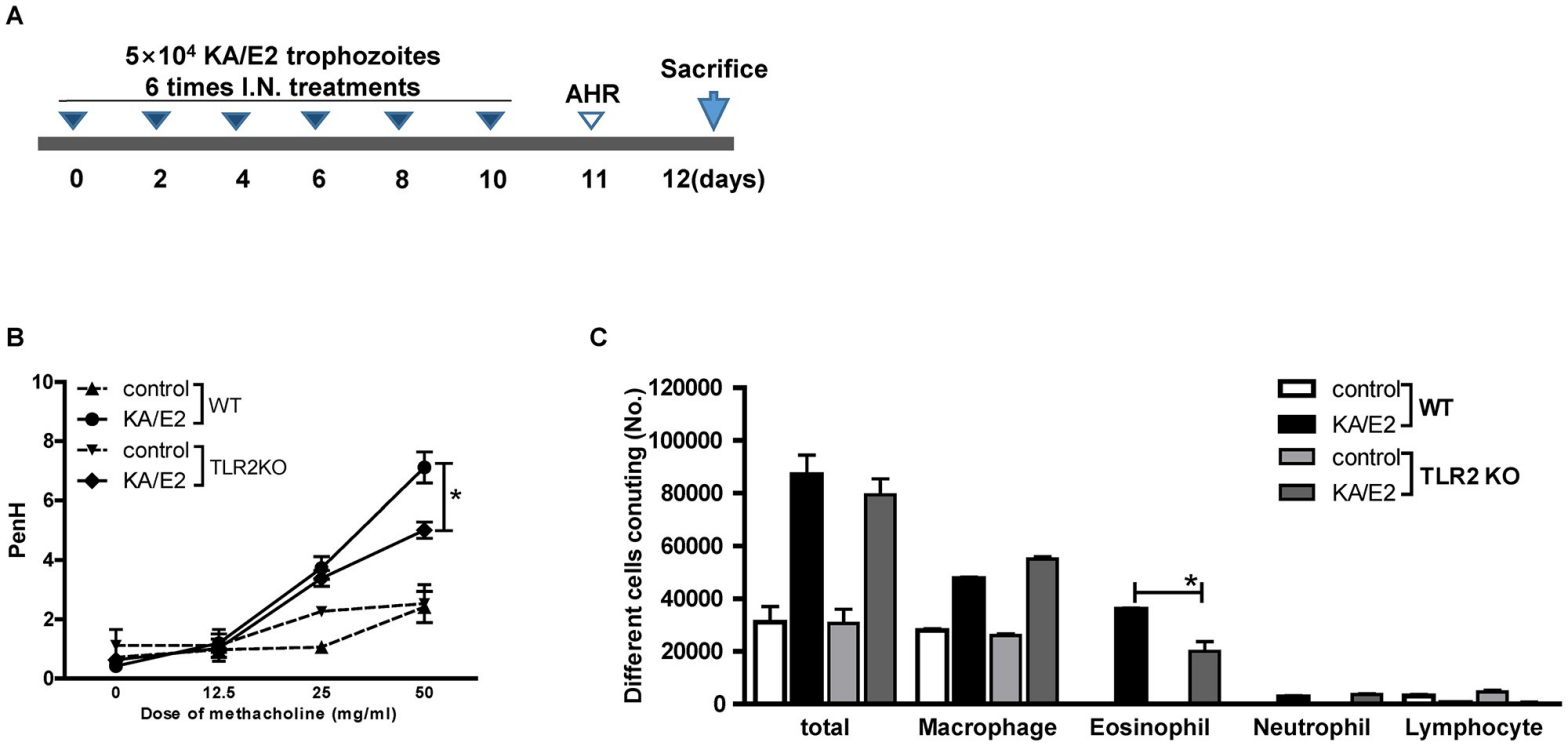
KA/E2 induced allergic airway inflammation via TLR2. Lung inflammation was induced via intranasal inoculation of 4x10^5^ KA/E2 trophozoites (A). Airway resistance values in response to methacholine (0 to 50 mg/ml) were compared between WT and TLR2 KO mice treated with KA/E2 (B). Differential inflammatory cells were counted in BAL from WT or TLR2 KO mice using a microscope (C). (n = 5 mice/group, 3 independent experiments, *; *p* < 0.05).

**Fig 3:**
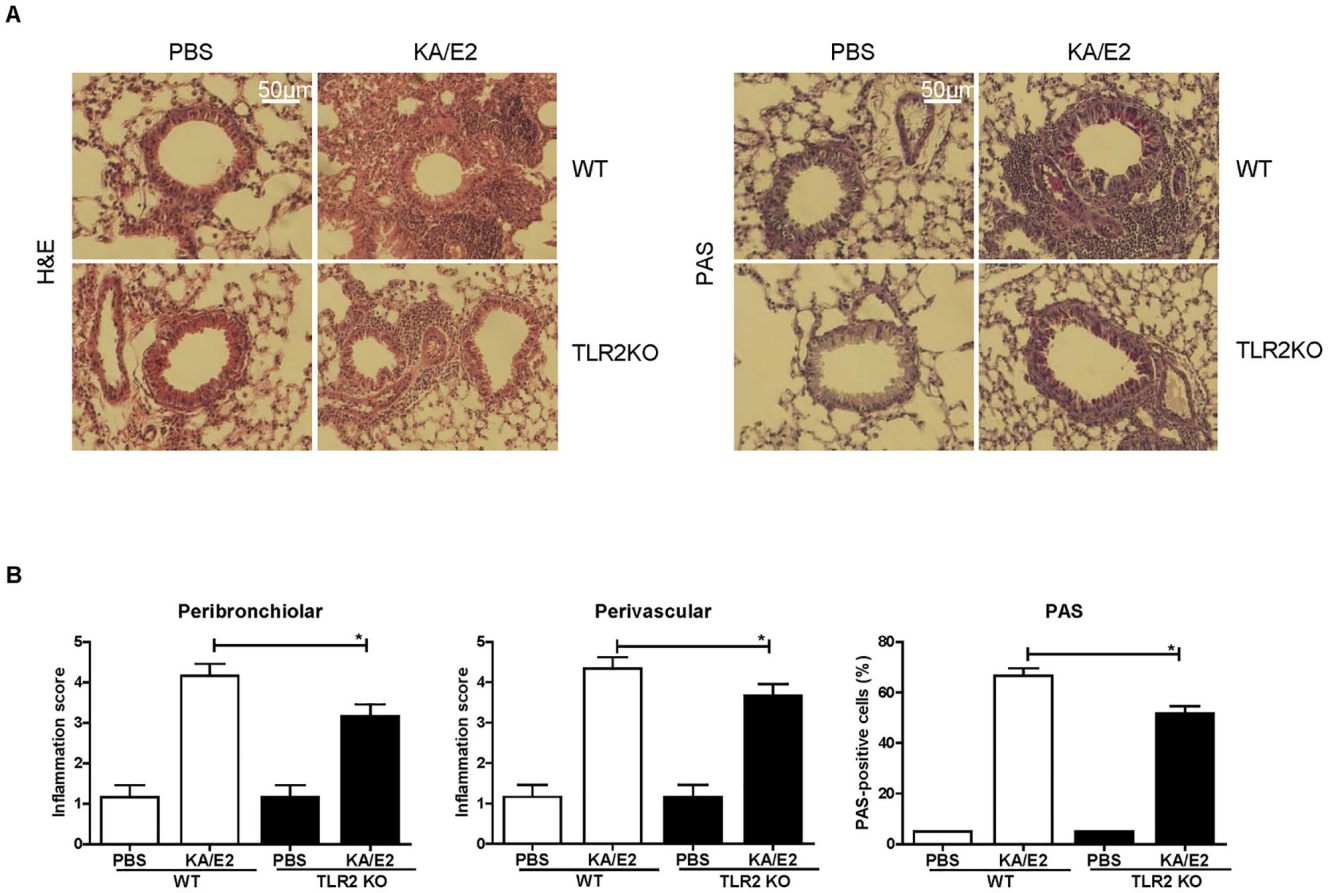
Comparison of the histopathological changes after KA/E2 treatment in WT and TLR2 KO mice. Tissue inflammation was observed in the images of lung sections after H&E or PAS staining (A). Representative inflammation scoring and PAS-positive cells (mean ± SEM). A grade of 0 indicates no detectable inflammation and a grade 4 indicates high percentages of airways and blood vessels in section cuffing by inflammatory cells (0 = normal tissue; 1 = <25%; 2 = 25–50%; 3 = 51–75%; 4 = >75%). Severity scoring was based on the thickness of the bronchi or vessels surrounding inflammatory cells (0 = no cells; 1 = 1–3; 2 = 4–6; 3 = 7–9 cells thick; 4 = 10 or more cells thick). Means of the *p*-value were calculated for comparison to the control (n = 5 mice/group, 3 independent experiments, *; *p* < 0.05).

**Fig 4:**
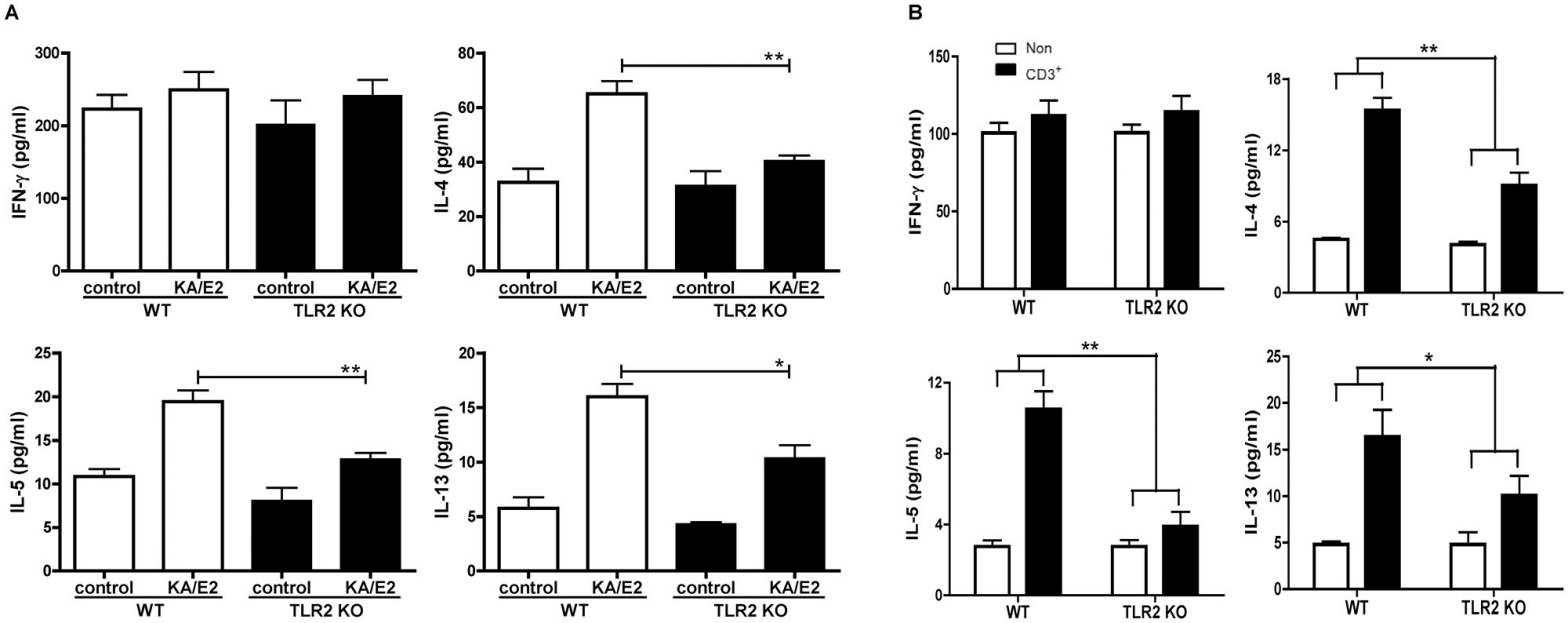
Comparison of the Th2 cytokine expression level after treatment with KA/E2 in WT and TLR2 KO mice. Th2 related cytokine (IL-4, IL-5, and IL-13) concentrations in the BALF (A) and culture supernatants of CD3-stimulated lymphocytes from LLN (B) were determined using ELISA. The plates were read at 450 nm on a standard ELISA reader, VICTOR 3 (Each *p*-value was determined using *t*-test methods compared to the control) (n = 5 mice/group, 3 independent experiments, *p < 0.05, **p < 0.01).

To determine whether TLR2 mediates allergic airway inflammation induced by KA/E2, we inoculated the KA/E2 trophozoites in WT and TLR2 KO mice (Fig 2A). The airway resistance values decreased after methacholine administration in the KA/E2-treated TLR2 KO group compared to the treated WT group (Fig 2B). In addition, the number of inflammatory cells, particularly eosinophils, in BALF was significantly lower in TLR2 KO mice than in WT mice (Fig 2C). Compared to WT mice with KA/E2 exposure, the TLR2 KO mice displayed reduced immune cell infiltration around the bronchial tracts, mucin production, and hyperplasia of lung epithelial cells and goblet cells (Fig 3). Finally, the levels of IL-4, -5, and -13 in BALF and lung-draining lymph nodes from TLR2 KO mice were lower than those in WT mice (Fig 4). These data indicate that TLR2 affected allergic airway inflammatory responses induced by KA/E2 intranasal inoculation in vivo.

### KA/E2 ES-P–treated DCs induce the differentiation of Th2 cytokines via TLR2

**Fig 5:**
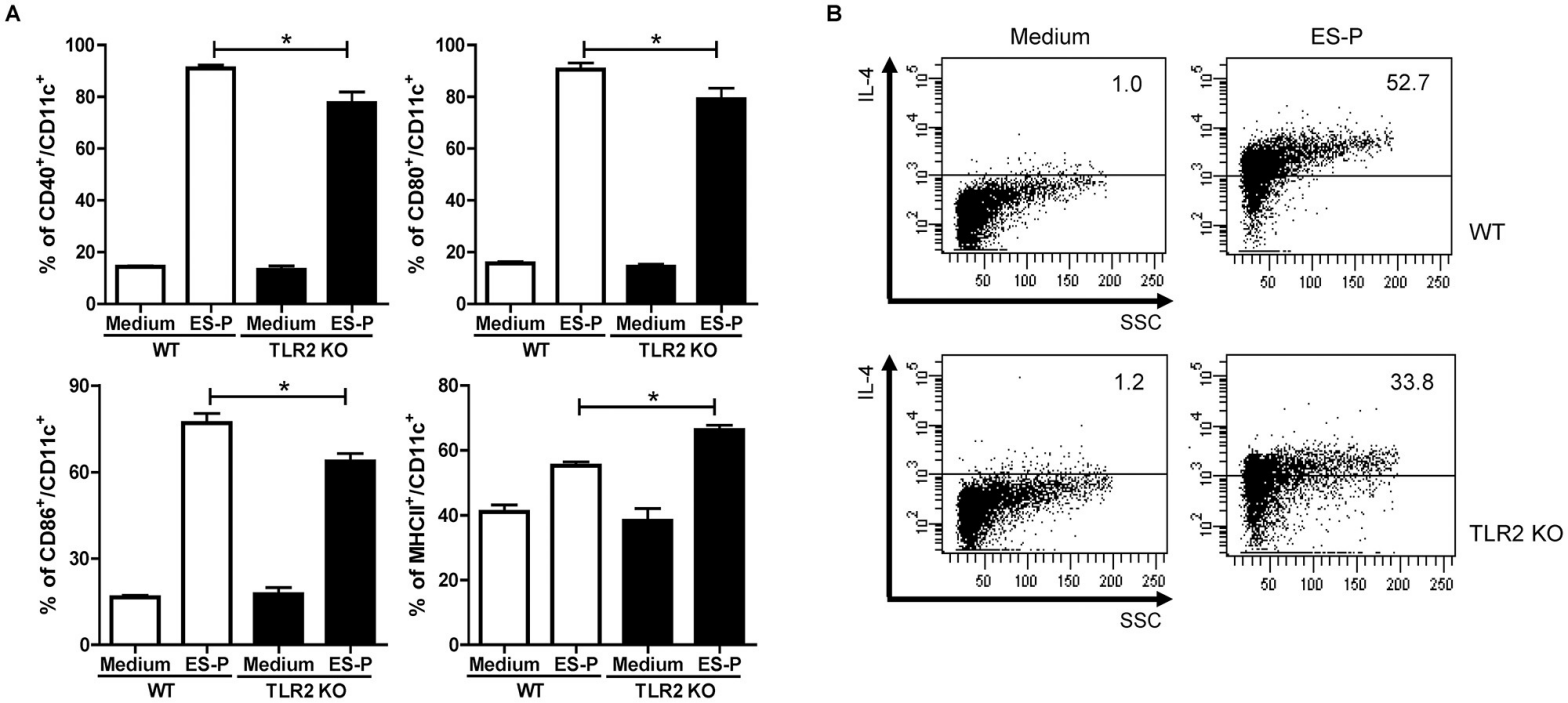
Comparison of DC surface marker activation and the production of Th2-related cytokine expressing cells. Activated BMDCs surface marker (A) and IL-4 expressing T cells (B) were compared between the WT and TLR2 KO groups. (n = 3/group, 3 independent experiments, *; *p* < 0.05, **; *p* < 0. 01, ***; *p* < 0.001).

BMDCs from WT and TLR2 KO mice were treated with KA/E2 ES-P and the expression levels of the surface markers, CD40, 80, 86, and MHCII, were determined using FACS analysis. The ES-P-induced DC markers were found to be significantly reduced under the TLR2 knockout condition (Fig 5A). ES-P-treated BMDCs from WT and TLR2 KO mice were co-cultured with naïve T cells. Owing to TLR2 knockout, the expression of the Th2-related cytokine, IL-4, in cells was markedly lower than that in cells in the ES-P–treated WT group (Fig 5B). These data indicate that TLR2 plays a role in the activation of BMDCs and production of Th2 cells following KA/E2 ES-P treatment in vitro.

### TLR2 knockout reduces the expression of pro-inflammation-related genes in primary lung cells

**Fig 6:**
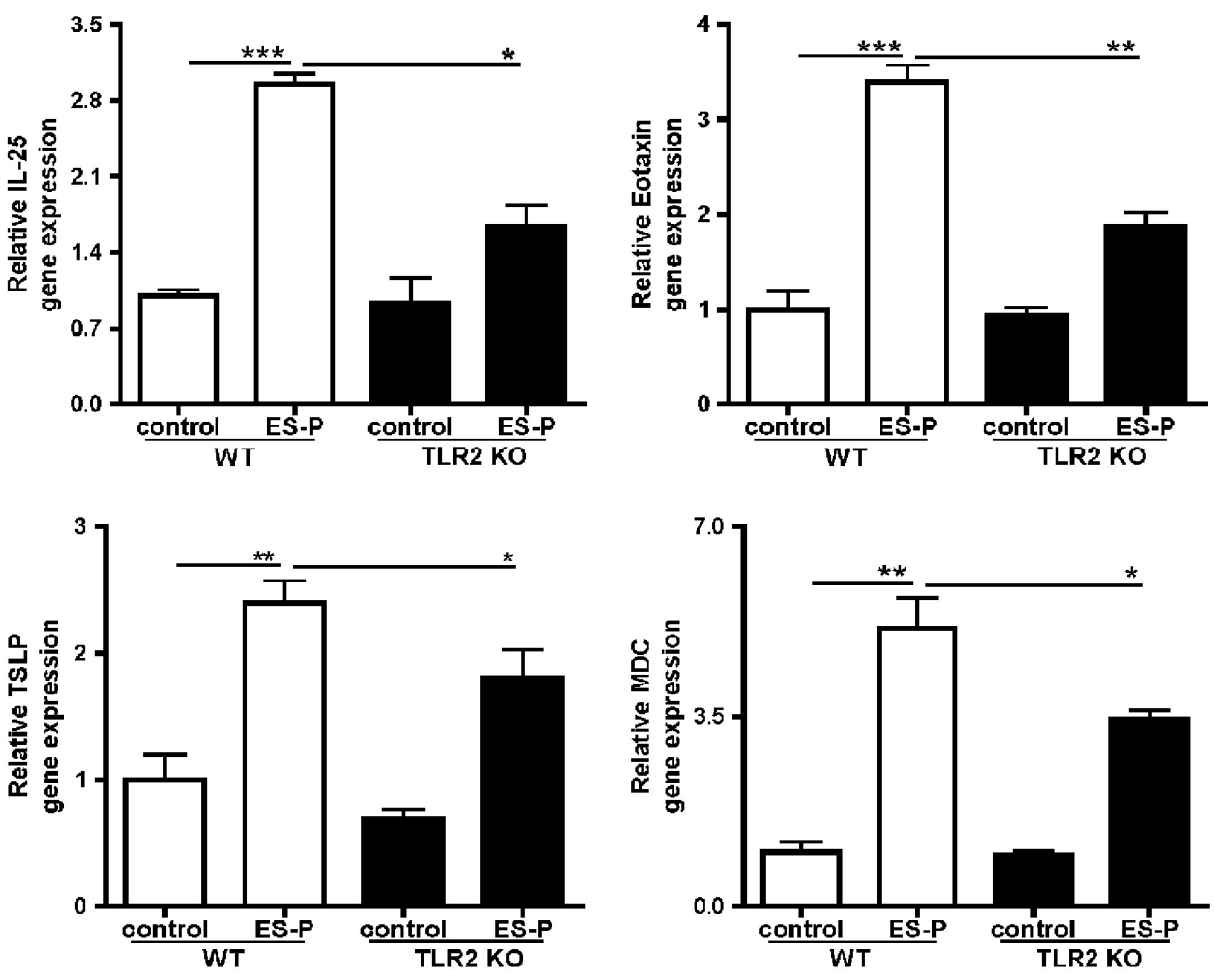
Evaluation of the inflammatory-related gene expression owing to KA/E2 ES-P in primary lung cells from WT and TLR2 KO mice. Assessment of the expression of inflammatory response related genes in primary lung cells from WT and TLR2 KO mice. (n = 3/group, 3 independent experiments, *; *p* < 0.05, **; *p* < 0. 01, ***; *p* < 0.001).

The mRNA expression levels of eotaxin, TARC, and TSLP in primary lung cells obtained from WT and TLR2 KO mice were determined via real-time PCR and the mRNA levels of the pro-inflammation-related genes in ES-P-treated TLR2 KO cells were significantly decreased (Fig 6). Figs 1 and 3 are the datasets used to determine the interaction between TLR2 and KA/E2 in vitro, especially in lung epithelial cells.

## Discussion

Airway epithelial cells express pattern recognition receptors, such as TLRs, retinoic acid-inducible gene-I-like receptors, nucleotide-binding oligomerization domain-like receptors, C-type lectin receptors, protease-activated receptors, and purinergic receptors [17,18]. They recognize PAMPs from inhaled microbes, parasites, and allergens, and damage-associated molecular patterns released from dying or damaged cells. Upon recognition of PAMPs or damage-associated molecular patterns, pattern recognition receptors activate downstream signaling, which promotes the release of pro-inflammatory cytokines and chemokines, including IL-6, IL-8, eotaxin, MDC, TSLP, IL-25, IL-33, TARC, and GM-CSF [19]. According to several studies, allergens exhibit intrinsic protease activities, and some proteases from infectious agents, parasites, and fungi have been identified as potent [6,20]. A previous study demonstrated that the protease activity of KA/E2 ES-P induces allergic airway inflammation in mice by inducing a Th2 response via protease-activated receptor 2 [3]. *Acanthamoeba* comprises a serine family of proteins with protease activity; however, it also contains many other antigens. In a previous clinical study, an *Acanthamoeba*-positive reaction was closely related to several pollen allergens, especially willow tree, poplar, elm, oak, velvet grass, and cockroach. IgE antibodies of patients who tested positive in the skin-prick test reacted strongly to the 15 kDa ES-P. In addition, these antigens reacted in patients who tested positive to pollens in the skin-prick test [7]. The 15 kDa antigen was identified as *Acanthamoeba* KA/E2 profilin (Ac-PF), suggesting that Ac-PF, which has no serine protease activity, elicited allergic airway inflammatory responses in mice [4]. In the present study, to determine the association between KA/E2 trophozoites or ES-P and pattern recognition receptors, we confirmed the inflammation-related gene expression levels. Based on the results, the expression of related genes decreased in most antagonist pretreatment groups; however, no significant difference was found. Among them, significant expression differences were observed only in the TLR2 or TLR9 pretreated groups, especially in the group pretreated with the TLR2 antagonist (Fig 1). TLRs play important roles in innate and adaptive immunity, balancing the immune response and inducing immune tolerance. Among them, TLR2 is the most widely expressed member of the TLR family on the surfaces of immune cells, macrophages, neutrophils, monocytes, and dendritic cells. The expression of TLR2 in lymphocytes has been confirmed in both mice and humans. The occurrence or acute exacerbation of asthma is reported to be associated with infection by different pathogens [17,18]. Therefore, the occurrence or acute exacerbation of asthma after pathogen infection is speculated to be closely related to the TLR2-mediated mechanism [19].

Based on the results of preliminary experiments with TLRs antagonists, we estimated the degree of disease underlying repeated exposure to KA/E2 trophozoites using TLR2 KO mice (Fig 2A). Airway resistance in the TLR2 KO group was found to be lower than that in the WT group (Fig 2B). In addition, the number of inflammatory cells, particularly of eosinophils, in BALF was significantly lower in TLR2 KO mice than in WT mice (Fig 2C). Fewer pathological changes were observed in TLR2 KO mice than in WT mice after KA/E2 exposure (Fig 3). The expression levels of Th2 related cytokines (IL-4, -5, and -13) in the BALF and lung-draining lymph nodes from the TLR2 KO group were lower than those from the WT group (Fig 4). These data indicate that TLR2 plays a role in inducing airway inflammatory responses in vivo following KA/E2 exposure. BMDCs from TLR2 KO mice displayed lower levels of activated surface markers than WT BMDCs following KA/E2 ES-P stimulation (Fig 5A). In addition, ES-P-treated TLR2 KO BMDCs induced the production of Th2 cytokine-expressing cells to a lesser extent (Fig 5B). Finally, in primary lung cells lacking TLR2, the expression levels of the pro-inflammation-related genes for ES-P were lower than those in WT cells (Fig 5). As shown in Figs 4 and 5, TLR2 is involved in the expression of inflammatory-related genes following KA/E2 ES-P stimulation in vitro.

In conclusion, TLR2 is involved in the occurrence and development of *Acanthamoeba* KA/E2–induced asthmatic airway inflammation. A detailed mechanistic study is needed; however, these results suggest that antigens other than the protease contained in KA/E2 induce Th2 and eosinophilic airway inflammatory responses via TLR2 in mice.
